# Immune Profiling of Syngeneic Murine and Patient GBMs for Effective Translation of Immunotherapies

**DOI:** 10.3390/cells10030491

**Published:** 2021-02-25

**Authors:** Jasneet Kaur Khalsa, Khalid Shah

**Affiliations:** 1 Center for Stem Cell Therapeutics and Imaging, Brigham and Women’s Hospital, Harvard Medical School, Boston, MA 02115, USA; Jasneet_Khalsa@DFCI.HARVARD.EDU; 2 Department of Neurosurgery, Brigham and Women’s Hospital, Harvard Medical School, Boston, MA 02115, USA; 3 Harvard Stem Cell Institute, Harvard University, Cambridge, MA 02138, USA

**Keywords:** glioblastoma, immune-profiling, RNA sequencing, CyTOF, tumor-microenvironment

## Abstract

Immunotherapy for brain tumors remains elusive, unlike many other cancer types for which it is one of the most promising therapeutic options. Recent studies have comprehensively profiled the immune-landscape of the highly malignant brain tumor, glioblastoma (GBM) in patients and identified novel immune-modulatory targets. However, given that pre-clinical exploration of potential novel therapeutics is primarily performed in immune-competent mice, it is vital to compare the immune-profiling data obtained from syngeneic mouse GBM models with GBM patient samples. This will pave the way for utilizing appropriate clinically relevant mouse GBM models for evaluating novel immune-therapies in pre-clinical settings. Recent brain tumor immune-profiling studies using state-of-the-art time of flight cytometry (CyTOF) analysis compared different human and mouse GBM types and reported immunological distinctions amongst these mouse models. These studies also contrast the immune phenotype of brain tumor patients with commonly used pre-clinical immune-competent mouse models. In this perspective, we provide the outcomes of very recent brain tumor immune-profiling studies and their implications on designing and translating unique, tumor-subtype specific therapeutics.

## 1. Introduction

Glioblastoma (GBM) is a highly invasive, incurable form of brain cancer and despite recent advances in therapeutic strategies, the prognosis of patients with GBM remains poor, with a median survival of 12–19 months. Immune therapies have revolutionized the field of cancer therapeutics in the past decade, however, their successful translation in GBM is feeble, attributed, at least in part, to the unique challenge posed by the blood brain barrier. Till recently, the brain was considered an immune-privileged site, however, with recent technological advancements, high dimensional characterization of the immune compartment of brain has been elucidated [1]. New discoveries unraveling the involvement of genetics in the development and progression of GBM have broadened our knowledge; yet, its implication in the immune landscape in GBM patients is elusive [2]. Furthermore, comprehensive comparison immune landscape of patient GBM with pre-clinical GBM models in immunocompetent mice is non-existent. Two recent publications [3,4] evaluated the composition of different immune cell populations in GBM patients using RNA sequencing on sorted populations and CyTOF analysis. As pre-clinical validations of immune therapies is performed in mice, we analyzed brain tumor tissues of 4 genetically distinct pre-clinical GBM mouse models, as well as for GBM patients, for the abundance and composition of immune cells in these models and discovered immunological inert and active types.

## 2. Role of Technological Advancements in Understanding the Tumor Micro-Environment

Single cell analysis afforded by flow cytometry has become a cornerstone for identifying and characterizing immune cell subsets, but this is limited by the number of markers that can be studied concurrently. An advanced version that combines mass spectrometry and flow cytometry, called mass cytometry or cytometry by time of flight (CyTOF), can be utilized to simultaneously study 100 parameters in a single sample [5]. Amir et al. [6] presented for the first time, how CyTOF and viSNE, a visualization tool based on t-SNE (the Distributed Stochastic Neighbor Embedding (t-SNE) algorithm), could be utilized for high-throughput analysis of leukemia. Since then, a high volume of studies have advanced our understanding of the immune landscape in many cancer types. In our recently published study, we performed a comprehensive tumor micro-environment analysis using a combination of RNA sequencing, CyTOF and histopathological staining to understand the leukocyte landscape of primary brain tumors [7]. Our findings reveal that microglia and tumor associated macrophages (TAMs) comprise up to 80% of immune cells isolated from tumor tissues of GBM patient samples, whereas T cells add up to 15%, with the presence of a very limited number of B and NK cells. These findings were also corroborated by Friebel et al. [3] in their recently published study.

Primary brain tumors, such as GBM, are initiated in the brain, whereas metastatic brain tumors are initiated elsewhere in the body and spread to the brain [8]. Friebel et al. [3] investigated the leukocyte composition in tumor tissues from GBM patients and compared them with metastatic tumor invasions in the brain. Since metastatic brain tumors do not have the same cellular composition as primary brain tumors, it is not surprising that their leukocyte composition is distinct [9]. Friebel [3] reported that GBMs have an enormous population of activated microglia, while metastatic tumors are characterized by T cells and monocyte-derived macrophages. The study further compares isocitrate dehydrogenase (IDH) wild type (WT) and IDH mutant GBMs. Mutations in IDH are known to occur as an early oncogenic event in glioma genesis often resulting in increased methylation [10]. IDH wild-type GBMs are aggressive and have the worst prognosis as compared to their mutant (IDH-mutant) counterparts [11]. With the limited pool of IDH mutant GBM samples, no significant distinction between IDH WT and IDH mutant GBMs has been observed. TAMs also have distinct subsets in IDH WT and mutant GBMs [12], thus emphasizing the importance of underlying genetic makeup of these GBM subsets. However, for the development of therapies targeting these specific subsets and for their clinical translation, equivalent analysis in mouse GBM models is indispensable.

**Figure 1:**
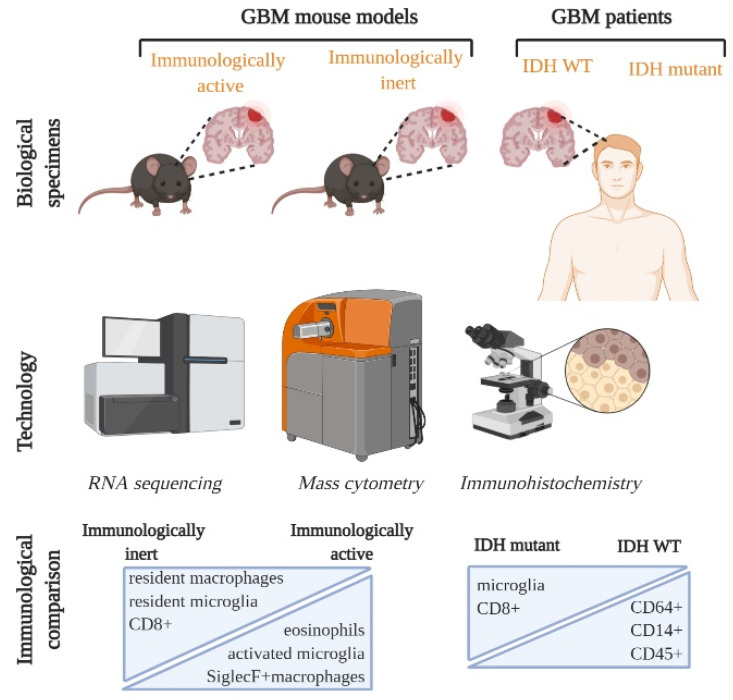
Single cell characterization of Immune landscape in GBM mouse models and patients. GBM mouse models were divided into immunologically active and inert based on RNA sequencing and GBM patients were categorized into 2 groups based on their IDH (isocitrate dehydrogenase) status: IDH WT and IDH mutant. Combining technological advances, such as RNA sequencing, CYTOF and immunohistochemistry, has allowed multi-parametric mapping of previously unexplored immune cell subsets. Immunologically inert mouse models and IDH mutant GBM patient tumors have numerous microglia and CD8+ populations. On the other hand, immunologically active and IDH WT patient GBMs are characterized by more leukocytes and activated microglia. GBM: glioblastoma; IDH: isocitrate dehydrogenase.

In our recently published study, we performed a systematic CyTOF analysis with corroborative IHC and RNA sequencing analysis on commonly available GBM mouse models and compared these analyses with GBM patient samples [7]. We first identified how microglia populations changed phenotype from resting to activated, along with an infiltration of other leukocytic populations when the naïve brain was compared with the tumor bearing brain. We followed this up with comparing genetically distinct mouse models: CT2A, GL261, Mut3 and 005, and identified key myeloid and lymphoid immune cell populations. Bulk RNA sequencing analysis revealed that mouse models could be separated into immunologically inert and active types based on the frequency of immune-related transcripts present in the tumor tissue. More specifically, our CyTOF analysis indicated that Siglec F+ macrophages and eosinophils were reduced in the immunologically inert mouse models, while exhausted CD8 T cells and resident macrophages were numerous [7] (Figure 1). Interestingly, Friebel et al. observed similar differences between their IDH WT and IDH mutant GBM patient samples (Figure 1). It would be interesting to explore if a similar composition can be found in patients with both few and numerous immune cells in the tumor micro-environment (TME). Our studies report compositional similarities in GBM mouse models and patient samples which can serve as an avenue for targeting and testing GBM subtype-specific therapeutics.

## 3. New Insights with Potential for Therapeutic Interventions

While comparing genetically distinct syngeneic GBM models, one interesting population that differed in these mouse models was eosinophils, which have been reported in GBM and many other cancers [13]. Eosinophils can trigger anti-tumor immune responses and inhibit the progression of GBM by enhancing T cell homing and polarizing macrophages [14]. High pre-treatment numbers of eosinophils in the peripheral blood of patients and temozolomide (TMZ) -induced post-surgery eosinophilia correlate with a better prognosis and improved survival in GBM patients [15,16]. Little is known about the immune-modulatory role of eosinophils in the tumor micro-environment and our study provides pertinent mouse models and an attractive approach to investigate and target this population in GBM. Further studies comparing eosinophils in metastatic versus primary tumors would shed more light on the role of eosinophils in primary and metastatic tumors both in patient tumor and syngeneic tumor settings.

As tumor resection is the primary intervention for GBM patients, it is imperative to understand the consequences of tumor debulking on the immune-phenotype of the tumor to optimally administer immune-therapeutic modalities, with the potential to further modulate the immune response. Previously, we developed a syngeneic orthotopic mouse GBM-model of tumor resection and showed that tumor debulking results in a substantial reduction in myeloid-derived suppressor cells (MDSCs) and simultaneous recruitment of CD4/CD8 T cells into the resection bed [17]. More importantly, we showed that tumor resection results in an increase in T cells and SiglecF^+^ macrophages in an immunosuppressive GBM mouse model [7]. This immunological signature mirrors immunologically active mouse models that have more SiglecF^+^ macrophages, along with fewer resident macrophages. It is essential to understand the baseline immune status of a resected tumor prior to the administration of any therapeutic intervention that further modifies the immune landscape. 

While assessing the immune phenotype of tumor tissues isolated from different syngeneic GBM mouse models and the tissue samples obtained during patient GBM tumor debulking, we identified the 005 GBM model that correlated most closely with patient GBM samples. Moreover, GL261, the most commonly used mouse model for pre-clinical immunotherapy studies, showed the presence of fewer antigen presenting cells (APC) APCs and more T cells than GBM patients. This might explain the success of immune checkpoint inhibitors, such as anti-PD-1 antibodies, that target T cells in the GL261 mouse model [18], while this does not translate efficiently with the lack of demonstrable survival benefit in the phase III clinical trial, checkmate 143 [19]. Comparing immune-active and immune-inert mouse models for efficacy and immune modulation post-therapy with various checkpoint inhibitors, might provide a more realistic and broader applicability of the treatment. Incorporating tumor resection in pre-clinical immune-modulators studies in mouse models will further enhance the likelihood of translational success in GBM patients. 

## 4. Comparing Pre-Clinical Models with Clinical Data Draws Previously Unidentified Parallels

GBM presents as one of the two subtypes phosphate and tensin homolog(PTEN, epidermal growth factor receptor (EGFR), *ink4a/Arf* or p53, platelet derived growth factor (PDGF) mutant) that have distinct molecular profiles and present distinct clinical signatures [20]. Previous studies have shown that loss of the tumor suppressor PTEN is linked with immune-resistance, mediated in part by PDL-1 [21], and PTEN loss increases programmed death ligand (PDL)-1 in cancer [22]. Separately, mutations in the p53 tumor suppressor gene are frequently detected in GBMs [23]. Our recent studies also suggest that genetically distinct GBM mouse models with respect to PTEN and p53 status, have a distinct immune landscape. Friebel et al. also showed distinctions between the IDH1 WT and mutant GBM subtypes, with microglia in IDH1 WT GBM being more reactive with respect to CD14 and CD64 expression. It would be prudent to determine a correlation—and possibly, a causal relationship—between underlying genetic makeup of the GBM subtype and immune status so as to stratify patients for various immune therapies. The applicability of reported findings from metastasis patients would also increase if similar analyses is performed in mouse models of metastasis. RNA sequencing and IHC performed on patient-matched melanoma brain and extracranial metastases show differences in immune cell infiltration with fewer CD3 and CD8+ T cells, fewer monocytic lineage and dendritic cells being observed in melanoma brain metastasis. However, a similar study in mouse models has not yet been explored [24]. The generation of new, more clinically relevant mouse models and their immunological characterization will greatly benefit the pursuit of better immune-targeting therapies. 

As the bulk of GBM is comprised of TAMs, therapeutic interventions aimed at reinforcing anti-tumor immunity by targeting these macrophages along with reviving T cells with check point inhibitor therapy, might be beneficial. Interaction between PD-1 expressed on T cells and PD-L1 expressed on GBM cells result in the inhibition of T cell functioning, leading to tumor invasion. A great deal of research has been conducted on blocking the PD-1/PD-L1 axis in GBM and clinical trials with monoclonal antibodies targeting PD-1/PD-L1 are underway [25]. Colony stimulating factor (CSF), secreted by GBM cells, facilitates TAM recruitment [26] and treatment with CSF-1 receptor (CSF-1R) antagonists has resulted in reduced infiltration of TAMs, decreases in tumor volume and increased survival of mice [27,28]. A recent study reported suppression of GBM recurrence and better survival in mice treated with the CSF-1R inhibitor post radiation therapy [29]. This also resulted in a concomitant increase in the CD8/CD4 T cell ratio [30] and thus, a pre-clinical study combining CSF1R antagonist with checkpoint inhibitors such as anti-PD-L1 therapy is underway [31].

## 5. Conclusions

Despite the limited genetic diversity of mouse models, recent advancements in immune-profiling techniques have expanded our ability to appreciate the nuances of cellular phenotypes, ultimately enabling us to design better therapeutic interventions aimed at modifying the immune tumor-microenvironment. To be able to design better, more efficacious immune-therapeutic drugs, there is an urgent need for immune-competent pre-clinical mouse models that closely resemble patients in terms of their tumor micro-environment.
